# Supplementary material for: Enhanced Pharmaceutically Active Compounds Productivity from Streptomyces SUK 25: Optimization, Characterization, Mechanism and Techno-Economic Analysis
Source: Molecules. 2021 Apr 25;26(9):2510. doi: 10.3390/molecules26092510 (PMC8123281; doi:10.3390/molecules26092510)
Supplement: Supplementary file 1 [file molecules-26-02510-s001.zip › molecules-1162661-supplementary.pdf]

Supplementary data

Article

Enhanced pharmaceutically active compounds productivity from *Streptomyces* SUK 25; optimization; characterization, mechanism, and techno-economic analysis

Muhanna M. Al-Shaibani [1, 2], Radin Maya Saphira Radin Mohamed [1,*], Noraziah M. Zin [2,*], Adel Al-Gheethi [1,*], Mohammed Al-Sahari [1] and Hesham Ali El Enshasy [3,4]

Table S1:

The central composite design arrangement and responses for pharmaceutically active compounds production from *Streptomyces* SUK 25

Run	x_1	x_2	x_3	x_4	x_5	x_6	x_7	y_1		y_2		y_3	
								Actual	Predicted	Actual	Predicted	Actual	Predicted
1	0	7	28	140	1	3	0.5	0.0000	0.4613	0.0000	28.04	2.00	3.68
2	3	7	28	140	1	3	0.5	3.83	3.34	64.00	20.49	11.40	9.08
3	6	7	28	140	1	3	0.5	5.70	5.49	16.00	14.32	13.20	13.29
4	9	7	28	140	1	3	0.5	7.00	6.93	8.00	9.53	17.70	16.33
5	12	7	28	140	1	3	0.5	8.70	7.64	4.00	6.13	19.60	18.18
6	15	7	28	140	1	3	0.5	7.50	7.63	4.00	4.11	18.90	18.85
7	18	7	28	140	1	3	0.5	6.20	6.90	8.00	3.48	17.90	18.34
8	21	7	28	140	1	3	0.5	6.00	5.45	8.00	4.24	17.40	16.65
9	12	4	28	140	1	3	0.5	3.40	2.77	8.00	8.29	12.90	11.55
10	12	5	28	140	1	3	0.5	4.30	4.79	8.00	7.57	12.90	14.30
11	12	6	28	140	1	3	0.5	4.30	6.41	8.00	6.85	12.90	16.51
12	12	7	28	140	1	3	0.5	8.70	7.64	4.00	6.13	19.60	18.18
13	12	8	28	140	1	3	0.5	7.70	8.47	8.00	5.42	17.60	19.31
14	12	9	28	140	1	3	0.5	8.70	8.91	4.00	4.71	19.60	19.91
15	12	7	26	140	1	3	0.5	5.74	6.09	16.00	22.71	15.60	16.03
16	12	7	28	140	1	3	0.5	8.70	7.64	4.00	6.13	19.60	18.18
17	12	7	30	140	1	3	0.5	7.70	8.23	8.00	6.27	17.60	18.64
18	12	7	32	140	1	3	0.5	7.70	7.86	8.00	23.14	17.60	17.42
19	12	7	34	140	1	3	0.5	6.70	6.53	64.00	56.72	14.60	14.52
20	12	7	28	120	1	3	0.5	8.20	8.58	8.00	10.40	17.60	18.03
21	12	7	28	140	1	3	0.5	8.70	7.64	4.00	6.13	19.60	18.18

22	12	7	28	160	1	3	0.5	5.74	6.88	16.00	23.20	15.60	16.89
23	12	7	28	180	1	3	0.5	6.70	6.32	64.00	61.60	14.60	14.17
24	12	7	28	140	1	3	0.5	8.70	7.64	4.00	6.13	19.60	18.18
25	12	7	28	140	1.5	3	0.5	4.30	6.42	8.00	0.23	12.90	16.45
26	12	7	28	140	2	3	0.5	7.70	5.89	8.00	6.48	17.60	15.39
27	12	7	28	140	2.5	3	0.5	5.74	6.03	6.00	26.26	15.60	15.00
28	12	7	28	140	3	3	0.5	6.70	6.86	64.00	59.12	14.60	15.27
29	12	7	28	140	1	1	0.5	6.70	7.44	64.00	32.94	14.60	16.91
30	12	7	28	140	1	1.5	0.5	5.74	6.21	16.00	18.18	15.60	15.50
31	12	7	28	140	1	2	0.5	7.70	5.84	8.00	8.79	17.60	15.24
32	12	7	28	140	1	2.5	0.5	4.30	6.31	8.00	4.77	12.90	16.14
33	12	7	28	140	1	3	0.5	8.70	7.64	4.00	6.13	19.60	18.18
34	12	7	28	140	1	1	0.5	8.70	7.44	4.00	32.94	19.60	16.91
35	12	7	28	140	1	1	1	4.30	6.09	8.00	10.68	12.90	15.33
36	12	7	28	140	1	1	1.5	7.70	5.91	8.00	5.32	17.60	15.17
37	12	7	28	140	1	1	2	6.30	6.90	16.00	16.89	15.60	16.41

x_1 (Time) (Day), x_2 (pH), x_3 (Temperature) (°C), x_4 (Speed) (rpm), x_5 (Glucose) (g/L), x_6 (Mannitol) (g/L), x_7 (Asparagine) (g/L), y_1(Crude extracts) (mg/L), y_2MIC) (μg/mL), y_3IZ) (mm).

Table S2 The regression coefficient and their significance of the quadratic model for pharmaceutically active compounds production from *Streptomyces* SUK 25

**Term**	**Coefficient**			**Standard Error**			**F value**			**P-value**		
	y_1	y_2	y_3	y_1	y_2	y_3	y_1	y_2	y_3	y_1	y_2	y_3
**Model**	1.86	10.03	9.07	2.17	24.63	3.52	3.80	2.70	4.83	0.0026	0.0180	0.0005
x_1	2.49	11.90	6.48	0.7262	8.22	1.17	11.79	2.09	30.50	0.0024	0.1619	< 0.0001
x_2	3.07	1.79	4.18	0.7975	9.03	1.29	14.82	0.0392	10.53	0.0009	0.8448	0.0037
x_3	0.2232	-17.01	-0.7574	0.7601	8.61	1.23	0.0862	3.90	0.3801	0.7718	0.0608	0.5439
x_4	-1.13	-25.60	-1.93	0.8728	9.88	1.41	1.68	6.71	1.87	0.2084	0.0167	0.1850
x_5	-0.3909	-26.50	-1.45	0.6570	7.44	1.06	0.3540	12.69	1.87	0.5579	0.0017	0.1847
x_6	0.0994	13.41	0.6318	0.4911	5.56	0.7938	0.0409	5.81	0.6334	0.8415	0.0247	0.4346
x_7	-0.2710	8.03	-0.2516	0.7894	8.94	1.28	0.1179	0.8063	0.0389	0.7346	0.3789	0.8455
x_1^2	-4.42	-8.48	-7.23	1.00	11.36	1.62	19.37	0.5570	19.88	0.0002	0.4634	0.0002
x_2^2	-1.23	-0.0156	-1.68	1.07	12.09	1.73	1.33	1.668E-06	0.9445	0.2607	0.9990	0.3417
x_3^2	-1.92	-33.44	-3.37	1.33	15.10	2.16	2.07	4.90	2.44	0.1638	0.0375	0.1325
x_4^2	0.2132	-24.00	-1.61	1.19	13.47	1.92	0.0321	3.17	0.7045	0.8594	0.0886	0.4103
x_5^2	1.36	-26.15	1.33	1.22	13.78	1.97	1.25	3.60	0.4602	0.2763	0.0709	0.5046
x_6^2	1.70	-10.75	2.30	1.10	12.45	1.78	2.40	0.7460	1.68	0.1359	0.3971	0.2086
x_7^2	1.31	-19.03	1.59	1.42	16.04	2.29	0.8601	1.41	0.4819	0.3638	0.2481	0.4948

x_1 (Time) (Day), x_2 (pH), x_3 (Temperature) (°C), x_4 (Speed) (rpm), x_5 (Glucose) (g/L), x_6 (Mannitol) (g/L), x_7 (Asparagine) (g/L), y_1(Crude extracts) (mg/L), y_2MIC) (µg/mL), y_3(IZ) (mm).

Table S3 : Peaks absorbance values of FT-IR spectra of PHACs

Frequency Wave number cm^{-1}	Assignment
3345	N–H and O–H stretching
3257	N–H and O–H stretching
3083	
2901	Aromatic C–H stretching
1684	Phenyl group
1562	N–O stretch (ArNo2) & C–C stretching vibrations
1412	C–H deformations & C–C stretching vibrations
1343	
1245	O–H bending
1062	C–O stretch (Primary alcohol)
974	C–N stretch- C – Chlorine
815	Meta di substituted aromatic ring ortho-di substiuted aromatic ring due to –H, moving out of plane of the benzene ring
652	C– chlorine bonds

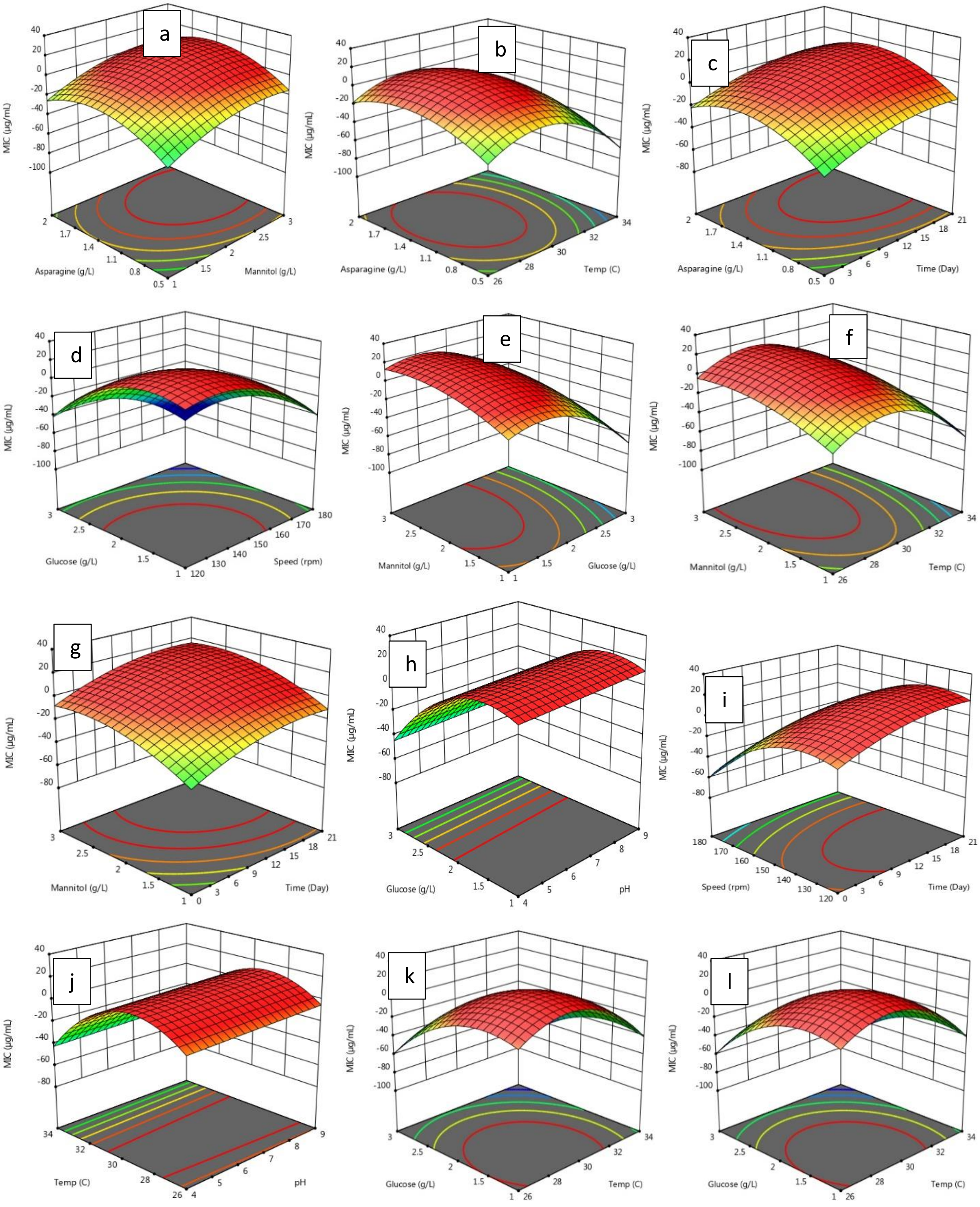

Figure S1. Three-dimensional response surface plot for interactions between x_1 (time) (day), x_2 (pH), x_3 (temperature) (°C), x_4 (speed) (rpm), x_5 (glucose) (g/L), x_6 (mannitol) (g/L), x_7 (asparagine) (g/L) and their effects on y_2 (MIC) (μg/mL).

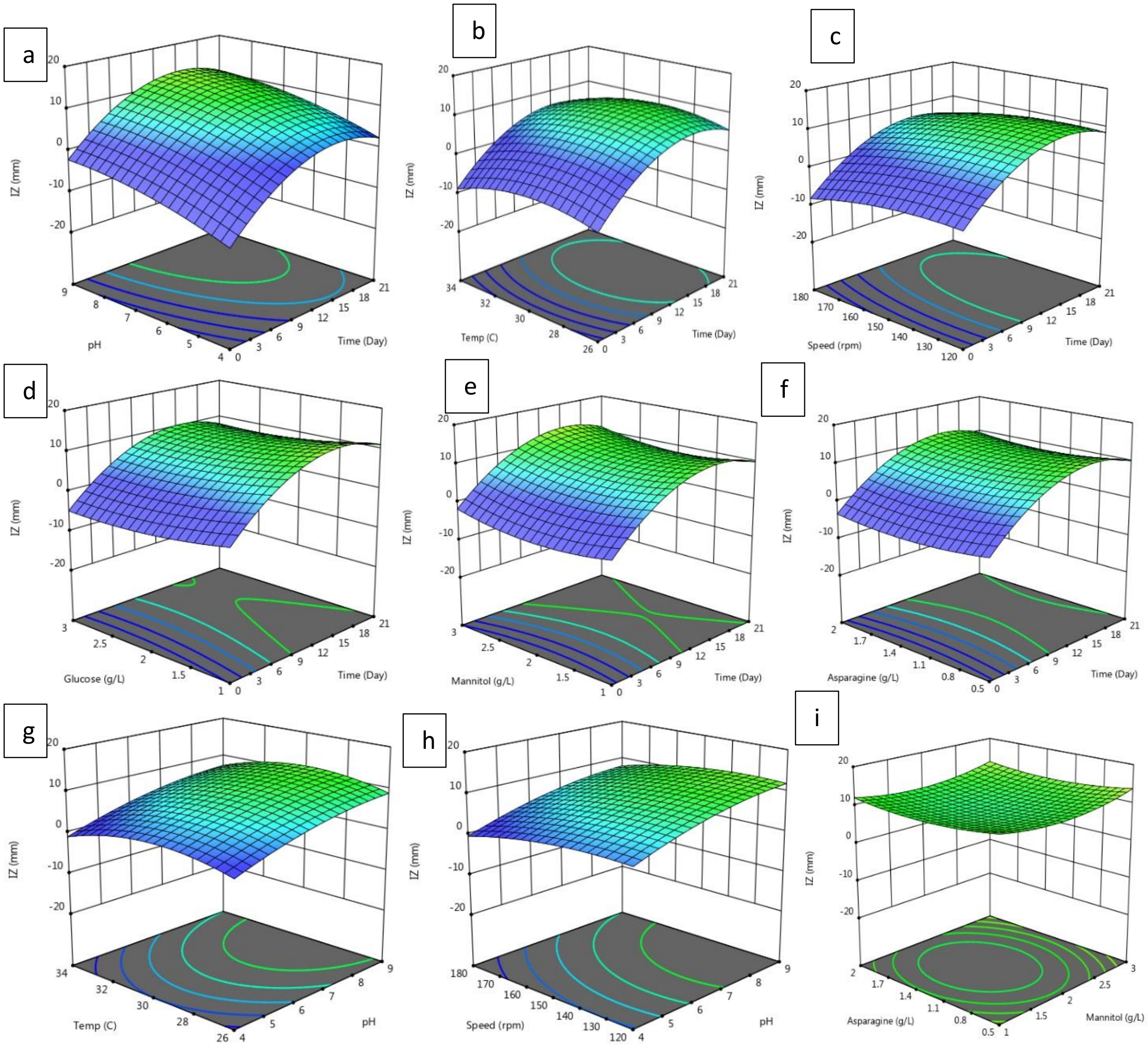

Figure S2: Three-dimensional response surface plot for interactions between x_1 (time) (day), x_2 (pH), x_3 (temperature) (°C), x_4 (speed) (rpm), x_5 (glucose) (g/L), x_6 (mannitol) (g/L), x_7 (asparagine) (g/L) and their effects on y_3IZ) (mm).
